# Unsaturated fatty acids suppress interleukin-2 production and transferrin receptor expression by concanavalin A-stimulated rat Iymphocytes

**DOI:** 10.1155/S0962935192000188

**Published:** 1992

**Authors:** Philip C. Calder, Eric A. Newsholme

**Affiliations:** 1 Cellular Nutrition Research Group Department of Biochemistry University of Oxford South Parks Road Oxford OXl 3QU UK

## Abstract

The proliferation of T-lymphocytes is dependent upon their ability to synthesize and secrete the cytokine, interleukin-2, and to express cell surface receptors for interleukin-2 and transferrin. We have previously reported that certain fatty acids inhibit mitogen-stimulated T-lymphocyte proliferation. We now report that unsaturated fatty acids decrease the concentration of interleukin-2 in the culture medium of such cells by up to 45%. This suggests that unsaturated fatty acids inhibit lymphocyte proliferation by suppressing interleukin-2 production. However, lymphocyte proliferation was only partially restored by addition of exogenous interleukin-2 to cell culture medium in the presence of unsaturated fatty acids, indicating that these fatty acids also affect other processes involved in the control of proliferation. Saturated fatty acids, which also inhibit lymphocyte proliferation, did not affect the interleukin-2 concentration in the culture medium suggesting a different mechanism for their action. Neither saturated nor unsaturated fatty acids affected the expression of the interleukin-2 receptor by mitogenstimulated lymphocytes. In contrast, unsaturated fatty acids decreased expression of the transferrin receptor by up to 50%. These observations suggest that the mechanism by which unsaturated fatty acids inhibit lymphocyte proliferation involves suppression of interleukin-2 production and of transferrin receptor expression. The mechanism for the inhibitory action of saturated fatty acids is not clear.


Mediators of Inflammation, 1, 107–112 (1992)

### Unsaturated fatty acids suppress interleukin-2 production and transferrin receptor expression by concanavalin A-stimulated rat lymphocytes

**Philip C. Calder**[CA] **and Eric A. Newsholme**

Cellular Nutrition Research Group, Department of Biochemistry, University of Oxford, South Parks Road, Oxford OX1 3QU, England.

[CA] Corresponding Author

**THE proliferation of T-lymphocytes is dependent upon their ability to synthesize and secrete the cytokine, interleukin-2, and to express cell surface receptors for interleukin-2 and transferrin. We have previously reported that certain fatty acids inhibit mitogen-stimulated T-lymphocyte proliferation. We now report that unsaturated fatty acids decrease the concentration of interleukin-2 in the culture medium of such cells by up to 45%. This suggests that unsaturated fatty acids inhibit lymphocyte proliferation by suppressing interleukin-2 production. However, lymphocyte proliferation was only partially restored by addition of exogenous interleukin-2 to cell culture medium in the presence of unsaturated fatty acids, indicating that these fatty acids also affect other processes involved in the control of proliferation. Saturated fatty acids, which also inhibit lymphocyte proliferation, did not affect the interleukin-2 concentration in the culture medium suggesting a different mechanism for their action. Neither saturated nor unsaturated fatty acids affected the expression of the interleukin-2 receptor by mitogen-stimulated lymphocytes. In contrast, unsaturated fatty acids decreased expression of the transferrin receptor by up to 50%. These observations suggest that the mechanism by which unsaturated fatty acids inhibit lymphocyte proliferation involves suppression of interleukin-2 production and of transferrin receptor expression. The mechanism for the inhibitory action of saturated fatty acids is not clear.**

**Key words:** Fatty acid, Interleukin-2, Lymphocyte proliferation

### Introduction

T-lymphocytes are activated by interaction with stimuli such as antigens, mitogens or antibodies directed against cell surface structures. Activation of T-cells results in production of interleukin-2 (IL-2) and appearance on the cell surface of receptors for IL-2 and for transferrin. Although the expression of IL-2 receptors precedes that of transferrin receptors,[1,2] the appearance of both types of receptor is crucial for subsequent proliferation of the activated T-cell.[2-4] Proliferation of such cells also requires the continued production of IL-2.[5,6] Therefore, a decrease in the rate of IL-2 production or in the expression of the receptors for IL-2 or for transferrin would be expected to decrease the rate of T-lymphocyte proliferation. Indeed, defects in IL-2 production result in a decreased *in vitro* response to mitogens,[5-7] the loss of high affinity IL-2 receptors leads to termination of T-cell proliferation *in vitro*,[8-10] and blocking of transferrin receptors with monoclonal antibodies inhibits DNA synthesis and cell division.[2,4]

We have shown that a number of fatty acids at physiological concentrations can inhibit the proliferation of mitogen-stimulated rat lymphocytes in culture.[11] All nine fatty acids tested inhibited the response to concanavalin A (Con A) but the extent of inhibition was dependent upon the fatty acid concentration used, the time of fatty acid addition to the culture medium and the duration of exposure of the cells to the fatty acid.[11] Generally, unsaturated fatty acids were more inhibitory than saturated fatty acids; the greatest inhibition of proliferation was caused by eicosapentaenoate (20:5 *n*-3) or arachidonate (20:4 *n*-6) and the least inhibition was caused by myristate (14:0) or palmitate (16:0).

The mechanism by which fatty acids affect lymphocyte proliferation is unknown. Therefore, the effects of various fatty acids on some of the known requirements for proliferation of mitogen-stimulated rat lymphocytes in culture (i.e. IL-2 production and expression of receptors for IL-2 and for transferrin) were investigated. The fatty acids used were myristate, palmitate, stearate (18:0), oleate (18:1 *n*-9), linoleate (18:2 *n*-6), α-linolenate

© 1992 Rapid Communications of Oxford Ltd

(18:3 *n*-3), arachidonate, eicosapentaenoate and docosahexaenoate (22:6 *n*-3).

### Materials and Methods

*Materials:* These were obtained from sources as described previously.[11] CTLL-2 and G2 cell lines, recombinant human IL-2 (Cetus) and the IL-2 receptor blocking antibody (NDS 63) were generous gifts from Dr Maggie Dallman, Nuffield Department of Surgery, John Radcliffe Hospital, Headington, Oxford. Monoclonal antibodies (MRC OX-21, MRC OX-26, MRC OX-39) and fluorescein isothiocyanate-labelled rabbit anti-mouse IgG (RAM-FITC) were generous gifts from the MRC Cellular Immunology Unit, Sir William Dunn School of Pathology, University of Oxford, Oxford. Fatty acid–bovine serum albumin (BSA) complexes were formed as described previously.[11]

*T-lymphocyte proliferation assay:* Male Wistar rat cervical lymph node cells were isolated, purified and cultured as described elsewhere.[11] Briefly, the cells were cultured at 37°C in an air/$CO_2$ atmosphere (19:1) in 96-well microtitre culture plates (approx. $5 \times 10^5$ cells/well and a final culture volume of 200 $\mu$l) in a HEPES-buffered RPMI medium supplemented with 10% (v/v) foetal calf serum, 2 mM glutamine, 5 $\mu$g/ml Con A, 100 $\mu$M fatty acid (added as a complex with BSA) and antibiotics (100 units/ml streptomycin and 200 units/ml penicillin). In some experiments (see Results) the cell culture medium also contained recombinant human IL-2 (20 units/ml). After 48 h, [6-$^3$H]-thymidine (0.2 $\mu$Ci/well) was added and the cells were cultured for a further 18 h. The cells were then harvested onto glass fibre filters which were washed and dried using a Skatron Cell Harvester. [$^3$H]-Thymidine incorporation was measured by liquid scintillation counting and was used to indicate cell proliferation.[12]

*Assay for IL-2 in culture media:* The concentration of IL-2 in lymphocyte culture media was determined by bioassays which used the IL-2-dependent murine CTLL-2[13] and rat G2[14] cell lines. The cell lines were maintained in a HEPES-buffered RPMI medium supplemented with 10% foetal calf serum, 2 mM glutamine, 25 $\mu$M 2-mercaptoethanol and recombinant human IL-2. Prior to their use in the bioassays, CTLL-2 and G2 cells were collected by centrifugation and washed three times in IL-2-free medium.

After culture of lymphocytes for various times in the conditions described above, the medium was removed, serially diluted two-fold and 100 $\mu$l aliquots transferred to microtitre plate wells. CTLL-2 or G2 cells ($1 \times 10^4$/100 $\mu$l) were added to each well. The plates were incubated for 18 h at 37°C and then [6-$^3$H]-thymidine was added (0.5 $\mu$Ci/well). After a further 6 h incubation the cells were harvested onto glass fibre filters, washed and dried; the incorporation of [$^3$H]-thymidine into DNA was measured. Recombinant human IL-2 was used to construct a standard curve of thymidine incorporation against IL-2 concentration. In some experiments the response of the G2 cells to IL-2 was blocked using NDS 63, an antibody which blocks the rat IL-2 receptor.[15] Preliminary experiments established that an NDS 63 concentration of 100 $\mu$g/ml could completely block the response of the G2 cell line to IL-2 (data not shown).

*Analysis of IL-2 and transferrin receptor expression:* Lymphocytes were cultured as described above, but at a concentration of $5 \times 10^6$ cells/well and a total culture volume of 2 ml in 24-well culture plates. After 48 h, the cells were removed and washed twice in $Ca^{2+}$- and $Mg^{2+}$-supplemented phosphate-buffered saline (PBS), pH 7.2, containing 0.1% BSA and 10 mM sodium azide. Approximately $10^6$ cells were incubated for 20 min at 4°C with monoclonal antibodies to the IL-2 receptor (MRC OX-39) or the transferrin receptor (MRC OX-26). Incubation with a monoclonal antibody to the human C3b activator protein (MRC OX-21) was used as a negative control. The cells were then washed twice with PBS and incubated for 20 min at 4°C with RAM-FITC. After washing twice with PBS, the cells were suspended in 'FACS-Fix' (2% formaldehyde in PBS) and examined using a Becton Dickinson FACScan fluorescence-activated cell sorter (FACS). Fluorescence data were collected on $1 \times 10^4$ viable cells, using forward light scatter. The distribution of fluorescently-labelled cells was computed using standard methods.

### Results

*Fatty acid inhibition of IL-2 production:* IL-2 was not detectable in the medium of lymphocytes cultured in the absence of Con A (Fig. 1). However, after stimulation of the cells by Con A, the IL-2 concentration in the culture medium was markedly increased (Fig. 1); it reached a maximum concentration of approximately 8 units/ml after 24 h of culture and remained high until at least 48 h (Fig. 1). The presence of BSA in the cell culture medium did not affect the IL-2 concentration (Fig. 1). Addition of myristate, palmitate or stearate to the culture medium did not affect the IL-2 concentration (Table 1). In contrast, unsaturated fatty acids decreased the IL-2 concentration in the cell culture medium by up to 45% after either 24 h or 48 h of culture (Table 1).

In addition to IL-2, the lymphocyte culture

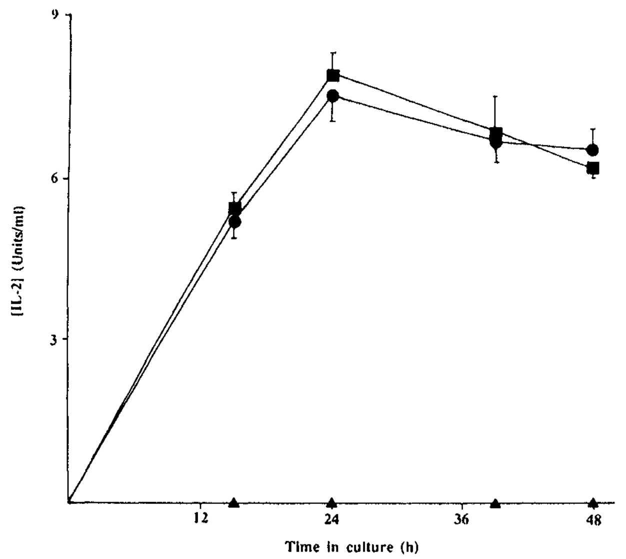

FIG. 1. The concentration of IL-2 in the culture medium of rat lymphocytes. Rat lymph node lymphocytes were cultured for in the absence (▲) or presence (■, ●) of Con A. In some cases the medium was also supplemented with 100 μM bovine serum albumin (●). At various times the medium was removed, serially diluted two-fold and the concentration of IL-2 determined using the CTLL-2 cell line (see Materials and Methods). Data are the mean ± SEM of twelve determinations.

medium will contain other cytokines, including interleukin-4 (IL-4). IL-2-dependent cell lines may also respond to IL-4. Therefore, the specificity of this bioassay for IL-2 was investigated by using the G2 cell line and the NDS 63 antibody, which blocks the IL-2 receptor on G2 cells. The response of G2 cells to IL-2 can be completely abolished using NDS 63 (data not shown). The IL-2 concentrations in lymphocyte culture media determined using the G2 cell line were similar to those obtained with the CTLL-2 cell line (Table 1). The specificity of these measurements for IL-2 was confirmed; IL-2 was not detected in the culture media if the G2-based bioassay was performed in the presence of 100 μg/ml NDS 63 (Table 1). Fatty acids themselves had no effect upon thymidine incorporation into either the CTLL-2 or G2 cell lines (data not shown).

*Partial reversal of fatty acid-induced inhibition of T-lymphocyte proliferation by IL-2:* If fatty acids cause inhibition of T-lymphocyte proliferation by lowering the concentration of IL-2, it would be expected that the addition of exogenous IL-2 would restore the proliferative response. Therefore, lymphocytes were cultured for 66 h in the presence of Con A, fatty acid and recombinant human IL-2 (20 units/ml) and thymidine incorporation measured over the final 18 h of culture. Addition of IL-2 to control lymphocyte cultures (i.e. no fatty acid added) had no effect on thymidine incorporation (Table 2). In the presence of saturated fatty acids, IL-2 addition did not affect thymidine incorporation (Table 2). In contrast, in the presence of unsaturated fatty acids, IL-2 addition increased thymidine incorporation (Table 2). However, the restoration of the proliferative response by IL-2 was only partial; fatty acids retained part of their inhibitory effect in the presence of IL-2 (Table 2).

*Effect of fatty acids on expression of the IL-2 and transferrin receptors:* The effect of fatty acids on the proportion of cells expressing receptors for IL-2 or transferrin was investigated using the MRC OX-39 and MRC OX-26 monoclonal antibodies, respectively; a monoclonal antibody to the human C3b activator protein (MRC OX-21) was used as a negative control. The proportion of cells staining positive with MRC OX-21 was always less than 8%, even following mitogenic stimulation.

The proportion of freshly prepared lymphocytes

**Table 1.** Effect of fatty acids on the concentration of IL-2 in the culture medium of Con A-stimulated rat lymphocytes

| Fatty acid | IL-2 concentration (units/ml) | | | |
|---|---|---|---|---|
| | 24 h[a] | 48 h[a] | 48 h[b] | 48 h[c] |
| None | 7.9 ± 0.4 (100) | 6.2 ± 0.2 (100) | 6.3 ± 0.2 (100) | <0.5 |
| *Saturated* | | | | |
| Myristate | 8.5 ± 0.4 (108) | 6.0 ± 0.2 (97) | 6.6 ± 0.2 (104) | <0.5 |
| Palmitate | 6.7 ± 0.5 (85) | 6.0 ± 0.3 (97) | 6.4 ± 0.4 (102) | <0.5 |
| Stearate | 6.6 ± 0.6 (84) | 5.6 ± 0.4 (90) | 6.2 ± 0.4 (98) | <0.5 |
| *Unsaturated* | | | | |
| Oleate | 6.3 ± 0.3[d] (80) | 4.7 ± 0.4[d] (76) | 5.0 ± 0.2[e] (79) | <0.5 |
| Linoleate | 6.2 ± 0.2[e] (78) | 3.0 ± 0.2[e] (48) | 3.8 ± 0.3[e] (60) | <0.5 |
| Linolenate | 6.3 ± 0.3[e] (79) | 3.8 ± 0.4[e] (61) | 3.6 ± 0.2[e] (57) | <0.5 |
| Arachidonate | 5.4 ± 0.2[e] (68) | 3.7 ± 0.3[e] (60) | 3.8 ± 0.5[e] (60) | <0.5 |
| Eicosapentaenoate | 4.3 ± 0.3[e] (54) | 3.6 ± 0.2[e] (58) | 3.4 ± 0.3[e] (54) | <0.5 |
| Docosahexaenoate | 4.4 ± 0.6[e] (56) | 4.1 ± 0.3[e] (66) | 4.2 ± 0.4[e] (67) | <0.5 |

Rat lymph node lymphocytes were cultured for 24 or 48 h in the presence of Con A and 100 μM fatty acid–albumin complexes. The medium was removed, serially diluted two-fold and the concentration of IL-2 determined using the CTLL-2[a] or G2[b,c] cell lines (see Materials and Methods). Assays using the G2 cell line were performed in the absence[b] or presence[c] of 100 μg/ml IL-2 receptor blocking antibody (NDS 63). Data are the mean ± SEM of 12[a] or 6[b,c] determinations. Statistical significance *vs.* control (i.e. no fatty acid addition): [d]$p < 0.01$, [e]$p < 0.001$. Numbers in parentheses are IL-2 concentration as a percentage of the control mean.

**Table 2.** Effect of fatty acids on Con A-stimulated lymphocyte proliferation in the presence and absence of added IL-2

| Fatty acid | Thymidine incorporation (dpm/well) | |
|---|---|---|
| | No addition | Exogenous IL-2 |
| None | 124 798 ± 5 415 (100) | 131 655 ± 2 540 (100) |
| *Saturated* | | |
| Myristate | 99 410 ± 2 011[a] (80) | 102 477 ± 4 636[b] (78) |
| Palmitate | 86 999 ± 1 275[b] (70) | 93 240 ± 3 204[b] (71) |
| Stearate | 54 931 ± 4 404[b] (44) | 65 109 ± 3 602[b] (49) |
| *Unsaturated* | | |
| Oleate | 47 407 ± 5 192[b] (38) | 67 421 ± 6 573[b,c] (51) |
| Linoleate | 37 968 ± 2 651[b] (30) | 78 542 ± 2 491[b,e] (60) |
| Linolenate | 52 256 ± 5 023[b] (42) | 80 472 ± 4 213[b,d] (61) |
| Arachidonate | 28 563 ± 3 173[b] (23) | 56 166 ± 3 566[b,e] (43) |
| Eicosapentaenoate | 16 896 ± 1 698[b] (14) | 54 070 ± 2 561[b,e] (41) |
| Docosahexaenote | 43 896 ± 4 704[b] (35) | 66 509 ± 2 500[b,d] (51) |

Rat lymph node lymphocytes were cultured in the presence of Con A and 100 $\mu$M fatty acid–albumin complexes. In some cases the medium was also supplemented with recombinant human IL-2 (20 units/ml). Proliferation was assessed by the incorporation of radioactive thymidine into DNA over the final 18 h of a 66 h culture period. Data are the mean ± SEM of six determinations. Statistical significance *vs.* control cells (i.e. no fatty acid addition): [a]$p < 0.01$, [b]$p < 0.001$; *vs.* no IL-2 addition: [c]$p < 0.05$, [d]$p < 0.01$, [e]$p < 0.001$. Numbers in parentheses are thymidine incorporation as a percentage of the control mean.

that were positive for IL-2 receptor expression was 2.5%. After culture for 48 h in the absence of Con A 8.4% of cells were IL-2 receptor-positive. The fluorescence profiles obtained for cells cultured in the presence of Con A are shown in Fig. 2; mitogenic stimulation resulted in an increase in IL-2 receptor-positive cells to 83.1% (Table 3). The presence of fatty acids did not alter the proportion of cells expressing the IL-2 receptor (Table 3).

The proportion of freshly prepared lymphocytes which expressed the transferrin receptor was 5.6%. After culture for 48 h in the absence of mitogen, 7.2% of cells were transferrin receptor-positive. Mitogenic stimulation resulted in an increase in transferrin receptor-positive cells to 32.9% (Table 3). Although the presence of saturated fatty acids in the culture medium did not change the percentage of cells that were transferrin receptor-positive (Table 3), the presence of unsaturated fatty acids resulted in a decrease in this percentage (Table 3). The proportion of lymphocytes expressing the transferrin receptor was decreased by 50% in the presence of some polyunsaturated fatty acids (Table 3).

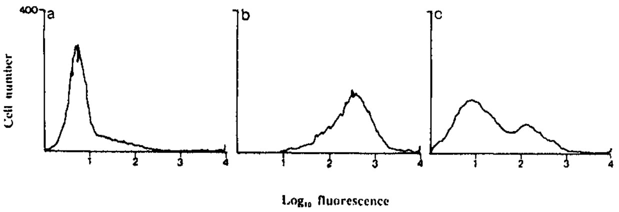

FIG. 2. Immunofluorescent profiles of Con A-stimulated lymphocytes. Rat lymph node lymphocytes were cultured in the presence of Con A. After 48 h the cells were washed and prepared for FACS analysis as described in Materials and Methods. The cells were incubated with (a) MRC OX-21 (anti-human C3b activator protein), (b) MRC OX-39 (anti-IL-2 receptor) or (c) MRC OX-26 (anti-transferrin receptor) and then stained with RAM-FITC. Stained cells were analysed in a FACScan analyser and the percentage of positive cells was determined by standard computerized methods.

**Table 3.** Effect of fatty acids on the expression of receptors for IL-2 and transferrin by Con A-stimulated rat lymphocytes

| Fatty acid | Con A | Percentage of receptor-positive cells | |
|---|---|---|---|
| | | IL-2 receptor | Transferrin receptor |
| None | – | 8.4 | 7.2 |
| None | + | 83.1 | 32.9 |
| *Saturated* | | | |
| Myristate | + | 87.7 | 33.3 |
| Palmitate | + | 80.3 | 31.6 |
| Stearate | + | 83.0 | 31.8 |
| *Unsaturated* | | | |
| Oleate | + | 88.4 | 20.6 |
| Linoleate | + | 81.4 | 24.0 |
| Linolenate | + | 85.0 | 16.7 |
| Arachidonate | + | 87.9 | 21.0 |
| Eicosapentaenoate | + | 87.6 | 20.0 |
| Docosahexaenoate | + | 87.6 | 18.1 |

Rat lymph node lymphocytes were cultured in the absence or presence of Con A and 100 $\mu$M fatty acid–albumin complexes. After 48 h the cells were collected, washed and incubated with monoclonal antibodies to the IL-2 receptor or transferrin receptor. After washing, the cells were incubated with RAM-FITC. After further washing, the cells were examined by FACS analysis. Data are the percentage of cells staining positive for each receptor, and are for a single, representative, cell preparation.

### Discussion

We have previously shown that fatty acids, in particular polyunsaturated fatty acids, inhibit Con A-stimulated proliferation of rat lymph node and human peripheral blood lymphocytes in culture.[11,16] Fatty acids also inhibit lipopolysaccharide-stimulated proliferation of rat lymph node lymphocytes.[17] In these studies, the fatty acids have been used at physiological concentrations and are presented to the cells as complexes with albumin, the means by which they are transported in the circulation. Fatty acids do not have a toxic effect since cell viability, as measured by trypan blue exclusion, was similar to that observed for cells cultured in the absence of exogenously-added fatty acids.[11] Furthermore, proliferation can be returned to normal if fatty acids are removed from the medium (P. C. Calder, unpublished data) or if a combination of fatty acids is added to the culture medium.[11] These observations indicate that inhibition of proliferation is not due to a detergent or other toxic effect of the fatty acids, and suggest that fatty acids exert their effects by interfering with the normal signalling mechanisms that control lymphocyte proliferation.

The proliferation of lymphocytes requires an increase in IL-2 secretion by activated T-cells and the expression of the IL-2 and transferrin receptors on the plasma membrane of such cells (see Introduction). In this study, we have demonstrated that unsaturated fatty acids lower the IL-2 concentration in the culture medium of mitogen-stimulated lymphocytes (Table 1). This suggests that the inhibitory effect of unsaturated fatty acids on mitogen-stimulated T-cell proliferation is mediated, at least in part, by the inhibition of IL-2 production. Addition of exogenous IL-2 did not completely restore the proliferative response in the presence of fatty acids (Table 2), suggesting that there is at least one further component of the fatty acid-induced inhibition and that this component is independent of the suppression of IL-2 production.

Unsaturated fatty acids did not affect IL-2 receptor expression but they did suppress expression of the transferrin receptor (Table 3). Expression of the transferrin receptor is induced by interaction between IL-2 and its receptor.[2] Because unsaturated fatty acids inhibited IL-2 production, it was possible that the inhibition of transferrin receptor expression was due to the decrease in IL-2 concentration. However, the inability of exogenous IL-2 to restore the proliferative response in the presence of fatty acids, indicates that fatty acids may interfere with transferrin receptor expression by a mechanism which is separate from their ability to inhibit IL-2 production. Thus, it appears that unsaturated fatty acids interfere with at least two distinct steps in the T-cell proliferative process: IL-2 production and transferrin receptor expression.

Saturated fatty acids did not affect the IL-2 concentration or the expression of receptors for IL-2 or transferrin (Tables 1, 3). How saturated fatty acids influence lymphocyte proliferation is not known at present.

The effects of unsaturated fatty acids upon the proliferation of T-lymphocytes are similar to those of prostaglandin $E_2$ ($PGE_2$), a well-documented inhibitor of mitogen-stimulated lymphocyte proliferation,[18–20] which inhibits both IL-2 production[21–23] and transferrin receptor expression[23], whilst not affecting IL-2 receptor expression.[23] The similarity of the effects of $PGE_2$ and unsaturated fatty acids suggests that the fatty acids may act via formation of immunosuppressive eicosanoids, such as $PGE_2$. However, we have recently presented strong evidence that the inhibitory effect of fatty acids upon lymphocyte proliferation is independent of eicosanoid production.[24]

Many of the events involved in lymphocyte activation and proliferation, including signal transduction and receptor expression, are membrane-associated and it is known that membrane functions are influenced by the fatty acid composition of the membrane phospholipids, probably via changes in membrane fluidity. It has recently been shown that during culture of activated lymphocytes, specific changes in fatty acid composition and membrane fluidity occur;[25] these changes may be an integral part of the proliferative response. Lymphocytes readily incorporate fatty acids into their lipids[26] and so the presence of an excess of one fatty acid in the culture medium may result in accumulation of that particular fatty acid in the phospholipids of the plasma membrane which would be expected to affect fluidity. Such a change in membrane fluidity could affect the signal transduction mechanisms which lead to synthesis of IL-2 and receptors. Furthermore, newly synthesized IL-2 must pass through the plasma membrane and newly synthesized transferrin receptors must be inserted into the plasma membrane. As such, changes in the membrane fluidity could cause the observed decreases in IL-2 production (Table 1) and transferrin receptor expression (Table 3), resulting in decreased lymphocyte proliferation (Table 2). In support of this, it has been shown that other agents which perturb membrane structure and/or fluidity also inhibit IL-2 production by T-lymphocytes. These agents include ethanol,[27] cyclosporin A,[28] 7,25-dihydroxycholesterol[29] and some carcinogens.[30,31] Cyclosporin A also suppresses transferrin receptor expression.[32,33]

In addition to an effect mediated via altered membrane fluidity, fatty acids could have a direct effect on transferrin receptor expression. The transferrin receptor is post-translationally acylated with palmitate[34] and it is known from work with a cell-free system that other fatty acids can substitute for palmitate.[35] The function of transferrin receptor acylation is not known. However, the importance of palmitoylation to membrane protein function is known from other systems. For example, the H-ras protein is palmitoylated and removal of the palmitate or its replacement with a myristoyl residue alters the affinity or anchoring of this protein to the membrane, suggesting that the acylation state can dramatically affect protein function.[36] Therefore, in the present study it is possible that the palmitic acid residue, which may be critical for transferrin receptor expression, has been replaced by other acyl groups; this could result in the down-regulation of transferrin receptor expression observed in the presence of unsaturated fatty acids (Table 3). Interestingly, the IL-2 receptor is not acylated and its expression was not affected by fatty acids.

### References

1. Hamilton TA. Regulation of Transferrin Receptor Expression in Concanavalin A Stimulated and Gross Virus Transformed Rat Lymphoblasts. *J Cell Physiol* 1982; **113:** 40–46.
2. Neckers L, Cossman J. Transferrin Receptor Induction in Mitogen-stimulated Human T-lymphocytes is Required for DNA Synthesis and Cell Division and is Regulated by Interleukin 2. *Proc Natl Acad Sci USA* 1983; **80:** 3494–3498.
3. Sutherland DR, Delia D, Schneider C, Newman RA, Kemehead J, Greaves MP. Ubiquitous Cell Surface Glycoprotein Related to Cell Proliferation is the Receptor for Transferrin. *Proc Natl Acad Sci USA* 1981; **78:** 4515–4519.
4. Mendelsohn J, Trowbridge I, Castagnola J. Inhibition of Human Lymphocyte Proliferation by Monoclonal Antibody to Transferrin Receptor. *Blood* 1983; **62:** 821–826.
5. Smith KA. T-cell Growth Factor. *Immunol Rev* 1980; **51:** 337–357.
6. Smith KA. Interleukin-2: Inception, Impact and Implications. *Science* 1988; **240:** 1169–1176.
7. Lopez-Botet M, Fontan G, Rodriguez MCG, de Landazuri MD. Relationship between IL-2 Synthesis and the Proliferative Response to PHA in Different Primary Immunodeficiencies. *J Immunol* 1982; **128:** 679–683.
8. Robb RJ, Munck A, Smith K. T cell growth factor receptors. Quantitation, Specificity and Biological Relevances. *J Exp Med* 1981; **154:** 1455–1474.
9. Cantrell D, Smith KA. Transient Expression of Interleukin 2 Receptors. Consequence for T Cell Growth. *J Exp Med* 1983; **158:** 1895–1911.
10. Cantrell D, Smith KA. The Interleukin-2 T-cell System: A new cell growth model. *Science* 1984; **224:** 1312–1316.
11. Calder PC, Bond JA, Bevan SJ, Hunt SV, Newsholme EA. Effect of Fatty Acids on the Proliferation of Concanavalin A-stimulated Rat Lymph Node Lymphocytes. *Int J Biochem* 1991; **23:** 579–588.
12. Szondy Z, Newsholme EA. The Effect of Various Concentrations of Nucleobases, Nucleosides or Glutamine on the Incorporation of [$^3$H]Thymidine into DNA in Rat Mesenteric Lymph Node Lymphocytes Stimulated by Phytohaemagglutinin. *Biochem J* 1990; **270:** 437–440.
13. Gillis S, Ferm MM, Ou W, Smith KA. T-cell Growth Factor: Parameters of production and a quantitative microassay for activity. *J Immunol* 1978; **120:** 2027–2032.
14. Schwinzer R, Schlitt HJ, Wonigeit K. Preferential Activation of CD45RA$^+$ Cells by a Monoclonal Antibody to the $\alpha/\beta$ T-cell Receptor. *Transplant Proc* 1991; **23:** 277.
15. Tillides G, Dallman MJ, Morris PJ. Mechanism of Action of Interleukin 2 Receptor (IL-2R) Monoclonal Antibody (MAb) Therapy: Target cell depletion or inhibition of function? *Transplant Proc* 1989; **21:** 997.
16. Calder PC, Newsholme EA. Polyunsaturated Fatty Acids Suppress Human Peripheral Blood Lymphocyte Proliferation and Interleukin-2 Production. *Clin Sci* 1992; in press.
17. Calder PC, Bond JA, Newsholme EA. Fatty Acid Inhibition of Lipopolysaccharide-stimulated B Lymphocyte Proliferation. *Biochem Soc Trans* 1990; **18:** 904–905.
18. Goldyne ME, Stobo JD. Immunoregulatory Properties of Prostaglandins and Related Lipids. *Crit Rev Immunol* 1981; **2:** 189–223.
19. Hwang D. Essential Fatty Acids and the Immune Response. *FASEB J* 1989; **3:** 2052–2061.
20. Kinsella JE, Lokesh B, Broughton S, Whelan J. Dietary Polyunsaturated Fatty Acids and Eicosanoids: Potential effects on the modulation of inflammatory and immune cells—an overview. *Nutrition* 1990; **6:** 24–44.
21. Rappaport RS, Dodge GR. Prostaglandin E Inhibits the Production of Human Interleukin-2. *J Exp Med* 1981; **155:** 943–948.
22. Walker C, Kristensen F, Bettens F, DeWeck AL. Lymphokine Regulation of Activated (G1) Lymphocytes. I. Prostaglandin $E_2$-induced inhibition of interleukin-2 production. *J Immunol* 1983; **130:** 1770–1773.
23. Chouaib S, Welte K, Mertelsmann R, Dupont B. Prostaglandin $E_2$ acts at two Distinct Pathways of T-lymphocyte Activation: Inhibition of interleukin-2 production and down-regulation of transferrin receptor expression. *J Immunol* 1985; **135:** 1172–1179.
24. Calder PC, Bevan SJ, Newsholme EA. The Inhibition of T-lymphocyte Proliferation by Fatty Acids is via an Eicosanoid-independent Mechanism. *Immunology* 1992; **75:** 108–115.
25. Anel A, Naval J, Gonzalez B, Torres JM, Mishal Z, Uriel J, Pineiro A. Fatty Acid Metabolism in Human Lymphocytes. I. Time-course changes in fatty acid composition and membrane fluidity during blastic transformation of peripheral blood lymphocytes. *Biochim Biophys Acta* 1990; **1044:** 323–331.
26. Anel A, Naval J, Gonzalez B, Uriel J, Pineiro A. Fatty Acid Metabolism in Human Lymphocytes II. Activation of fatty acid desaturase-elongase systems during blastic transformation. *Biochim Biophys Acta* 1990; **1044:** 332–339.
27. Kaplan DR. A Novel Method of Immunosuppression Mediated by Ethanol. *Cell Immunol* 1986; **102:** 1–9.
28. LeGrue SJ, Freidman AW, Kahan BD. Binding of Cyclosporine A by Human Lymphocytes and Phospholipid Vesicles. *J Immunol* 1983; **131:** 712–718.
29. Moog C, Luu B, Beck J, Italiano L, Bischoff P. Studies on the Immunosuppressive Properties of 7,25-Dihydrocholesterol. I. Reduction of interleukin production by treated lymphocytes. *Int J Immunopharm* 1988; **10:** 511–518.
30. House RV, Lauer LD, Murray MJ, Dean JH. Suppression of T-helper Cell Function in Mice Following Exposure to the Carcinogen 7,12 Dimethylbenz(a)anthracene and its Restoration by Interleukin-2. *Int J Immunopharm* 1987; **9:** 89–97.
31. Lyte M, Blanton RH, Myers MJ, Bick PH. Effect of *in vivo* Administration of the Carcinogen Benzo(a)pyrene on Interleukin-2 and Interleukin-3 Production. *Int J Immunopharm* 1987; **9:** 307–312.
32. Larsson EL. Cyclosporin A and Dexamethasone Suppress T Cell Response by Selectively Acting at Distinct Sites of the Triggering Process. *J Immunol* 1980; **124:** 2828–2833.
33. Lillehoj HS, Malek TR, Shevach EM. Differential Effect of Cyclosporin A on the Expression of T and B Lymphocyte Activation Antigens. *J Immunol* 1984; **133:** 244–250.
34. Omary MB, Trowbridge IS. Covalent Binding of Fatty Acid to the Transferrin Receptor in Cultured Human Cells. *J Biol Chem* 1981; **265:** 4715–4718.
35. Adam M, Rodriquez A, Turbide C, Larrick J, Meighen E, Johnstone RM. *In vitro* Acylation of the Transferrin Receptor. *J Biol Chem*, 1984; **259:** 15460–15463.
36. Deschenes RJ, Resh MD, Broach JR. Acylation and Prenylation of proteins. *Curr Opin Cell Biol* 1990; **2:** 1108–1113.

ACKNOWLEDGEMENTS. P.C.C. held a Nuffield Medical Fellowship. We would like to acknowledge the helpful advice of Dr Simon Hunt, Sir William Dunn School of Pathology and the generous gifts from Dr Maggie Dallman, Nuffield Department of Surgery.

**Received 22 November 1991; accepted 7 January 1992**

## References

[PDF_00646] Adam M., Rodriguez A., Turbide C., Larrick J., Meighen E., Johnstone R. M. (1984). In vitro acylation of the transferrin receptor.. J Biol Chem.

[PDF_00615] Anel A., Naval J., González B., Torres J. M., Mishal Z., Uriel J., Piñeiro A. (1990). Fatty acid metabolism in human lymphocytes. I. Time-course changes in fatty acid composition and membrane fluidity during blastic transformation of peripheral blood lymphocytes.. Biochim Biophys Acta.

[PDF_00619] Anel A., Naval J., González B., Uriel J., Piñeiro A. (1990). Fatty acid metabolism in human lymphocytes. II. Activation of fatty acid desaturase-elongase systems during blastic transformation.. Biochim Biophys Acta.

[PDF_00612] Calder P. C., Bevan S. J., Newsholme E. A. (1992). The inhibition of T-lymphocyte proliferation by fatty acids is via an eicosanoid-independent mechanism.. Immunology.

[PDF_00574] Calder P. C., Bond J. A., Bevan S. J., Hunt S. V., Newsholme E. A. (1991). Effect of fatty acids on the proliferation of concanavalin A-stimulated rat lymph node lymphocytes.. Int J Biochem.

[PDF_00593] Calder P. C., Bond J. A., Newsholme E. A. (1990). Fatty acid inhibition of lipopolysaccharide-stimulated B lymphocyte proliferation.. Biochem Soc Trans.

[PDF_00572] Cantrell D. A., Smith K. A. (1984). The interleukin-2 T-cell system: a new cell growth model.. Science.

[PDF_00570] Cantrell D. A., Smith K. A. (1983). Transient expression of interleukin 2 receptors. Consequences for T cell growth.. J Exp Med.

[PDF_00608] Chouaib S., Welte K., Mertelsmann R., Dupont B. (1985). Prostaglandin E2 acts at two distinct pathways of T lymphocyte activation: inhibition of interleukin 2 production and down-regulation of transferrin receptor expression.. J Immunol.

[PDF_00649] Deschenes R. J., Resh M. D., Broach J. R. (1990). Acylation and prenylation of proteins.. Curr Opin Cell Biol.

[PDF_00581] Gillis S., Ferm M. M., Ou W., Smith K. A. (1978). T cell growth factor: parameters of production and a quantitative microassay for activity.. J Immunol.

[PDF_00549] Hamilton T. A. (1982). Regulation of transferrin receptor expression in concanavalin A stimulated and Gross virus transformed rat lymphoblasts.. J Cell Physiol.

[PDF_00630] House R. V., Lauer L. D., Murray M. J., Dean J. H. (1987). Suppression of T-helper cell function in mice following exposure to the carcinogen 7,12-dimethylbenz[a]anthracene and its restoration by interleukin-2.. Int J Immunopharmacol.

[PDF_00598] Hwang D. (1989). Essential fatty acids and immune response.. FASEB J.

[PDF_00622] Kaplan D. R. (1986). A novel mechanism of immunosuppression mediated by ethanol.. Cell Immunol.

[PDF_00600] Kinsella J. E., Lokesh B., Broughton S., Whelan J. (1990). Dietary polyunsaturated fatty acids and eicosanoids: potential effects on the modulation of inflammatory and immune cells: an overview.. Nutrition.

[PDF_00637] Larsson E. L. (1980). Cyclosporin A and dexamethasone suppress T cell responses by selectively acting at distinct sites of the triggering process.. J Immunol.

[PDF_00624] LeGrue S. J., Friedman A. W., Kahan B. D. (1983). Binding of cyclosporine by human lymphocytes and phospholipid vesicles.. J Immunol.

[PDF_00640] Lillehoj H. S., Malek T. R., Shevach E. M. (1984). Differential effect of cyclosporin A on the expression of T and B lymphocyte activation antigens.. J Immunol.

[PDF_00634] Lyte M., Blanton R. H., Myers M. J., Bick P. H. (1987). Effect of in vivo administration of the carcinogen benzo(a)pyrene on interleukin-2 and interleukin-3 production.. Int J Immunopharmacol.

[PDF_00559] Mendelsohn J., Trowbridge I., Castagnola J. (1983). Inhibition of human lymphocyte proliferation by monoclonal antibody to transferrin receptor.. Blood.

[PDF_00626] Moog C., Luu B., Beck J. P., Italiano L., Bischoff P. (1988). Studies on the immunosuppressive properties of 7,25-dihydroxycholesterol--I. Reduction of interleukin production by treated lymphocytes.. Int J Immunopharmacol.

[PDF_00552] Neckers L. M., Cossman J. (1983). Transferrin receptor induction in mitogen-stimulated human T lymphocytes is required for DNA synthesis and cell division and is regulated by interleukin 2.. Proc Natl Acad Sci U S A.

[PDF_00643] Omary M. B., Trowbridge I. S. (1981). Covalent binding of fatty acid to the transferrin receptor in cultured human cells.. J Biol Chem.

[PDF_00603] Rappaport R. S., Dodge G. R. (1982). Prostaglandin E inhibits the production of human interleukin 2.. J Exp Med.

[PDF_00565] Robb R. J., Munck A., Smith K. A. (1981). T cell growth factor receptors. Quantitation, specificity, and biological relevance.. J Exp Med.

[PDF_00584] Schwinzer R., Schlitt H. J., Wonigeit K. (1991). Preferential activation of CD45RA+ cells by a monoclonal antibody to the alpha/beta T-cell receptor.. Transplant Proc.

[PDF_00563] Smith K. A. (1988). Interleukin-2: inception, impact, and implications.. Science.

[PDF_00562] Smith K. A. (1980). T-cell growth factor.. Immunol Rev.

[PDF_00556] Sutherland R., Delia D., Schneider C., Newman R., Kemshead J., Greaves M. (1981). Ubiquitous cell-surface glycoprotein on tumor cells is proliferation-associated receptor for transferrin.. Proc Natl Acad Sci U S A.

[PDF_00577] Szondy Z., Newsholme E. A. (1990). The effect of various concentrations of nucleobases, nucleosides or glutamine on the incorporation of [3H]thymidine into DNA in rat mesenteric-lymph-node lymphocytes stimulated by phytohaemagglutinin.. Biochem J.

[PDF_00587] Tellides G., Dallman M. J., Morris P. J. (1989). Mechanism of action of interleukin-2 receptor (IL-2R) monoclonal antibody (MAb) therapy: target cell depletion or inhibition of function?. Transplant Proc.

[PDF_00605] Walker C., Kristensen F., Bettens F., deWeck A. L. (1983). Lymphokine regulation of activated (G1) lymphocytes. I. Prostaglandin E2-induced inhibition of interleukin 2 production.. J Immunol.

